# Association between Smoking and Liver Fibrosis among Patients with Nonalcoholic Fatty Liver Disease

**DOI:** 10.1155/2019/6028952

**Published:** 2019-10-15

**Authors:** Hongjie Ou, Yaojie Fu, Wei Liao, Caixia Zheng, Xiaolu Wu

**Affiliations:** ^1^Department of Infectious Diseases, First Affiliated Hospital of Xiamen University, Xiamen, Fujian, China; ^2^Emergency Department, First Affiliated Hospital of Xiamen University, Xiamen, Fujian, China; ^3^Intensive Care Unit, Sun Yat-sen University Cancer Center, Guangzhou, China; ^4^Sun Yat-sen University Cancer Center, State Key Laboratory of Oncology in South China, Collaborative Innovation Center for Cancer Medicine, Guangzhou, China

## Abstract

**Objective:**

We aimed at analyzing the role of smoking in hepatic fibrosis in patients with nonalcoholic fatty liver disease (NAFLD) and at exploring the related risk factors.

**Methods:**

This was a cross-sectional study that included a total of 225 patients with NAFLD. Among them, 127 were nonsmokers and 98 were smokers. Liver significant fibrosis was diagnosed when the liver stiffness (LS) value was higher than 7.4 kPa. The diagnostic criterion for NAFLD was a controlled attenuation parameter (CAP) value of >238 dB/m. The CAP and LS values were measured using FibroScan.

**Results:**

FibroScan showed that the LS value in the smokers was significantly higher than that in the nonsmokers (10.12 ± 10.38 kPa vs. 7.26 ± 6.42 kPa, *P*=0.013). The proportions of patients with liver significant fibrosis and advanced liver fibrosis among the smokers were significantly higher than those among the nonsmokers (*P*=0.046). Univariate analysis showed that age, weight, high AST level, low PLT level, and smoking were the risk factors associated with liver fibrosis in the smokers with NAFLD while multivariate analysis showed that age (OR = 1.029, *P*=0.021), high AST level (OR = 1.0121, *P*=0.025), and smoking (OR = 1.294, *P*=0.015) were the independent risk factors associated with liver fibrosis in the patients with NAFLD. Moreover, high AST level (OR = 1.040, *P*=0.029), smoking index (OR = 1.220, *P*=0.019), and diabetes mellitus (OR = 1.054, *P*=0.032) were the independent risk factors for liver fibrosis among the smokers with NAFLD.

**Conclusion:**

This study showed that smoking was closely associated with liver fibrosis among the patients with NAFLD. For patients with NAFLD who smoke, priority screening and timely intervention should be provided if they are at risk of liver fibrosis.

## 1. Introduction

With the increase in the incidence of obesity and the associated metabolic syndrome, nonalcoholic fatty liver disease (NAFLD) has become an important cause of chronic liver disease [1]. Epidemiological studies have shown that 20% to 30% of individuals in Western countries develop NAFLD [1–3]. According to the definition of the American Association for the Study of Liver Diseases, NAFLD is a disease characterized by hepatic steatosis and lipid storage without excessive drinking history [2]. According to the change in the pathological degree, NAFLD can be divided into three stages: simple fatty liver (nonalcoholic fatty liver), nonalcoholic steatohepatitis, and cirrhosis.

NAFLD is closely related to metabolic syndrome [4]. Obesity, type 2 diabetes mellitus, and dyslipidemia are considered to be important risk factors for NAFLD [1, 4–6]. NAFLD is closely related not only to metabolic abnormalities but also to poor living behaviors [7–9]. All three abovementioned important risk factors for NAFLD are associated with unhealthy lifestyles. Therefore, NAFLD is generally considered to be a disease associated with an unhealthy lifestyle. Many studies have revealed that changes in unhealthy lifestyles can reduce the transaminase levels and improve NAFLD [10–13].

Smoking is a common poor living behavior in daily life. It can damage the antioxidant system [14, 15]. Although smoking can increase the risk for liver fibrosis and cirrhosis in patients with chronic hepatitis B (CHB) infection [16, 17], only a few studies have investigated the relationship between smoking and NAFLD. Suzuki et al. [18] reported that smoking is associated with high levels of alanine aminotransferase (ALT) in patients with NAFLD. Another study reported that smoking is an independent risk factor for NAFLD [19]. Although these authors have confirmed that smoking is associated with the occurrence of NAFLD, there is no research report on whether smoking promotes liver fibrosis in patients with NAFLD.

Hence, we enrolled smokers and nonsmokers with NAFLD and analyzed liver fibrosis among the smokers in this study. The risk factors for liver fibrosis were explored to provide medical evidence for screening and early diagnosis of liver fibrosis in smokers with NAFLD.

## 2. Methods

### 2.1. Subjects

This was a cross-sectional study that included a total of 225 patients with NAFLD. Among them, 127 were nonsmokers and 98 were smokers. All patients were recruited from the First Affiliated Hospital of Xiamen University from May 2015 to April 2018. Patients were included when they met the following criteria: diagnosis of NAFLD according to the diagnostic criterion of a controlled attenuation parameter (CAP) value of >238 dB/m according to previous recommendations and confirmation with ultrasonography [4, 20–22]. Conversely, patients were excluded when they met the following criteria: (1) use of medications that can induce hepatic steatosis (e.g., corticosteroids, estrogen, methotrexate, or amiodarone) within 6 months of study inclusion, (2) evidence of coinfection with hepatitis C, hepatitis D, or human immunodeficiency virus, (3) autoimmune liver disease, and (4) heavy alcohol consumption or alcohol abuse, defined as alcohol consumption of >10 g/day. The Institutional Review Board of the First Affiliated Hospital of Xiamen University approved the study. Each enrolled patient provided informed consent.

### 2.2. FibroScan Test

Liver fibrosis and steatosis were diagnosed on the basis of the liver stiffness (LS) and CAP values [23, 24]. These values were assessed by a professionally trained technician using FibroScan (Echosens, Paris, France) according to the manufacturer's instructions. The LS values were expressed in kilopascals and CAP values in decibels per meter. The ratio of the interquartile range (IQR) of the LS value to the median (IQR/M) was calculated as an indicator of variability. Only procedures with at least 10 valid measurements, a success rate of at least 60%, and an IQR/M ratio of <0.3 were considered reliable and then used for the analysis. The CAP value was measured only using validated measurement tools according to the same criteria used for the LS value and on the same signals, ensuring obtainment of a liver ultrasonic attenuation simultaneously and in the same volume of liver parenchyma as in the LS value. The median of the individual measurements was considered the final CAP value.

Among the patients with NAFLD, hepatic steatosis was diagnosed at the CAP values of >238 dB/m, according to previous recommendations [4, 20–22].

### 2.3. Patient Information Collection

Patient information, including demographic characteristics, physical examination, and laboratory test results, was collected. The demographic characteristics assessed included age, sex, and smoking history. Physical examination results, including height and weight, were recorded. Blood pressure was also measured after the FibroScan test. Laboratory test results, including the levels of platelet (PLT), serum aspartate aminotransferase (AST), and ALT, were collected in accordance with standard procedures. These laboratory test results were obtained using standard automated techniques within 14 days of the FibroScan test. Smoking index = daily tobacco intake *∗* duration of smoking.

Blood pressure was measured using a standard mercury sphygmomanometer. All patients were asked to rest for at least 5 minutes before measurement. Each patient required at least three blood pressure measurements, with an interval of 1 minute each. The average value of the three measurements was used for the analysis.

### 2.4. Statistical Analysis

Continuous variables were expressed as means ± standard deviations and categorical variables as percentages. The chi-square test and *t*-test were used to detect whether differences between the two groups were statistically significant. Univariate and multivariate logistic regression analyses were used to explore the risk factors associated with liver fibrosis and advanced liver fibrosis in the patients with NAFLD. The Data Analysis and Quality Control Program for SPSS for Windows version 13.0 was applied for the statistical analysis.

## 3. Results

### 3.1. Demographic and Clinical Characteristics of the Patients

A total of 225 patients with NAFLD were enrolled in this study. Among these patients, 98 were smokers (smoking group) and 127 were nonsmokers (nonsmoking group). The proportion of male patients in the smoking group was significantly higher than that in the nonsmoking group (*P* < 0.001). The weight of the patients in the smoking group was higher than that of the patients in the nonsmoking group (*P*=0.037). The proportion of patients with diabetes mellitus in the nonsmoking group was significantly lower than that in the smoking group (*P*=0.012). The serum levels of ALT, AST, and PLT were comparable between the two groups, as shown in Table 1.

### 3.2. Comparison of Liver Fibrosis between the Two Groups

The liver conditions of fibrosis were compared (Table 2). The LS value of the smoking group was significantly higher than that of the nonsmoking group (10.12 ± 10.38 kPa vs. 7.26 ± 6.42 kPa, *P*=0.013). The proportion of patients with liver significant fibrosis and advanced fibrosis in the smoking group was significantly higher than that in the nonsmoking group (*P*=0.046).

### 3.3. Risk Factors Associated with Fibrosis in the Patients with NAFLD

The univariate and multivariate analyses were conducted to explore the risk factors associated with fibrosis among the patients with NAFLD. The results are shown in Table 3. The univariate analysis showed that age, weight, high AST level, low PLT level, and smoking were the risk factors associated with liver fibrosis in the smokers with NAFLD. Conversely, the multivariate analysis showed that age (OR = 1.029, *P*=0.021), high AST level (OR = 1.0121, *P*=0.025), and smoking (OR = 1.294, *P*=0.015) were the independent risk factors associated with liver fibrosis in the patients with NAFLD.

### 3.4. Clinical Characteristics of the Patients with NAFLD with and without Liver Fibrosis

To analyze the related factors for liver fibrosis in the smokers with NAFLD further, we subdivided the smokers into the fibrosis and nonfibrosis subgroups. The clinical characteristics of these two subgroups are shown in Table 4. The average age (*P*=0.032) and AST level (*P*=0.001) in the fibrosis group were significantly higher than those in the nonfibrosis group, while the PLT level was lower in the fibrosis group than in the nonfibrosis group (*P*=0.036). In addition, the proportion of patients with diabetes mellitus in the fibrosis group was significantly higher than that in the nonfibrosis group (*P*=0.041); the smoking index was significantly higher in the fibrosis group than in the nonfibrosis group (*P*=0.043).

### 3.5. Risk Factors Associated with Fibrosis in Smokers with NAFLD

We further analyzed the factors associated with liver fibrosis in the smokers with NAFLD, and the results are shown in Table 5. The univariate analysis showed that age, high AST level, low PLT level, smoking index, and diabetes mellitus were the risk factors for fibrosis among these patients. Conversely, the multivariate analysis indicated that high AST level (OR = 1.040, *P*=0.029), smoking index (OR = 1.220, *P*=0.019), and diabetes mellitus (OR = 1.054, *P*=0.032) were the independent risk factors for liver fibrosis among them.

## 4. Discussion

In this study, we confirmed that smoking is closely associated with NAFLD. Moreover, we further confirmed that it is closely related to liver fibrosis in NAFLD. The LS value of the smokers with NAFLD was significantly higher than that of the nonsmokers with NAFLD. Older age, high AST level, and smoking were found to be the independent risk factors for liver fibrosis in the patients with NAFLD. Conversely, high AST level, smoking index, and diabetes mellitus were determined to be the independent risk factors for liver fibrosis in the smokers with NAFLD. These results imply that smoking is not only associated with liver fibrosis in NAFLD but also increases the risk for liver fibrosis as the smoking index increases.

The pathogenesis of NAFLD is not fully understood [1]. A widely accepted conclusion is that NAFLD is a genetic-environment-metabolism-related disease [1, 2]. Consumption of food high in calorie and fructose, refined carbohydrates, and sugar-sweetened beverages has been associated with NAFLD [1]. Recently, several genetic modifiers of NAFLD have been identified [25–28]. Among them, the best-characterized genetic association was found with PNPLA3, which was initially identified from genome-wide association studies and confirmed in multiple cohorts [29–32]. Liver biopsy is the gold standard for the diagnosis of NAFLD [1]. However, it cannot be routinely used because of its invasiveness. Noninvasive techniques, such as the use of FibroScan and ultrasonography, are beginning to be used for the diagnosis of NAFLD [20, 22]. Their accuracy has been confirmed in many studies [2, 16, 20–22].

The toxic and harmful substances produced by smoking can damage the antioxidant system, including cytochrome P450 and inflammatory cytokines [33]. Although the effects of smoking on CHB infection and cirrhosis have been reported [17, 34, 35], there is limited information on the relationship between smoking and NAFLD. Hamabe et al. [19] reported that smoking is an independent risk factor for NAFLD. Suzuki et al. [18] reported that it is associated with high levels of ALT in patients with NAFLD. Herein, we found that smoking is an independent risk factor for liver fibrosis in NAFLD. For patients with NAFLD, timely smoking cessation education should be provided, and a liver fibrosis test is also necessary. However, we found a connection between smoking index and liver fibrosis among NAFLD patients. However, in this population, the smoking index and liver fibrosis grade did not show with dose response in our study. We further found that diabetes mellitus and the smoking index were the independent risk factors for fibrosis in the smokers with NAFLD. The relationship between smoking and diabetes mellitus has been well established. If smokers with NAFLD are diagnosed with diabetes mellitus, the possibility of liver fibrosis development may increase. In our study, we found that diabetes mellitus is an independent factor for liver fibrosis among NAFLD patients with smoking. Although we did not find that DM is an independent factor for liver fibrosis among all NAFLD patients, the reason may due to the relatively small patients enrolled in our study with only 12 patients diagnosed with diabetes mellitus in NAFLD without smoking. In addition, since smoking is also an independent risk factor for DM, this may impair the association of DM and liver fibrosis among all NAFLD patients.

This study has some limitations. First, the sample size of the study is relatively small. Second, the study data were collected from a single center. Given the cross-sectional nature of this study, prospective studies should be conducted to corroborate the conclusions. Multicenter clinical studies are also warranted to confirm our results for screening and early diagnosis of liver fibrosis in patients with NAFLD.

In conclusion, smoking is closely related to liver fibrosis in NAFLD. Older age, high AST level, and smoking are the independent risk factors for liver fibrosis in NAFLD. Conversely, high AST level, smoking index, and diabetes mellitus are the independent risk factors for liver fibrosis in smokers with NAFLD. For patients with NAFLD, priority screening and timely intervention should be provided if they are found to have risk factors for liver fibrosis.

## Figures and Tables

**Table 1:** Baseline demographic and clinical characteristics by groups.

Variables	Nonalcoholic fatty liver disease		*P*
	Smoking	Nonsmoking	
Sample size	98	127	
Sex (F/M)	3/95	49/78	<0.001
Age (years)	45.77 ± 13.12	41.01 ± 11.55	0.004
Height (cm)	168.36 ± 6.47	166.96 ± 7.48	0.142
Weight (kg)	67.53 ± 12.50	63.98 ± 12.76	0.037
SBP (mm/Hg)	127.03 ± 12.52	129.09 ± 14.15	0.260
DBP (mm/Hg)	82.07 ± 7.85	83.66 ± 9.28	0.177
ALT	90.74 ± 72.34	80.49 ± 81.94	0.324
AST	67.84 ± 45.29	63.41 ± 40.67	0.444
PLT	216.11 ± 63.96	210.11 ± 60.91	0.476
T2DM (Y/N)	21/77	12/115	0.012

**Table 2:** Proportion of liver fibrosis and advanced fibrosis by groups.

Variables	Nonalcoholic fatty liver disease		*P*
	Smoking	Nonsmoking	
Sample size	98	127	
Liver stiffness value			0.046
<7.4 (kPa)	52	88	
7.4–9.8 (kPa)	22	16	
>9.8 (kPa)	24	23	
Liver stiffness value, kPa	10.12 ± 10.38	7.26 ± 6.42	0.013

**Table 3:** Risk factors associated with fibrosis in NAFLD patients.

Variables	Univariate analysis			Multivariate analysis		
	OR	95% CI	*P*	OR	95% CI	*P*
Sex	1.622	0.366–3.322	0.632			
Age	1.022	1.006–1.097	0.019	1.029	1.004–1.055	0.021
Height	0.936	0.875–1.001	0.055			
Weight	1.066	1.028–1.107	0.001			
SBP	1.014	0.958–1.073	0.632			
DBP	0.922	0.842–1.009	0.076			
ALT	0.998	0.994–1.002	0.339			
AST	1.009	1.001–1.017	0.020	1.012	1.002–1.061	0.025
PLT	0.991	0.985–0.996	0.001			
Smoking index	1.305	1.152–2.611	0.011	1.294	1.087–2.087	0.015
T2DM	1.022	0.998–1.525	0.062			

**Table 4:** Characteristics of NAFLD patients with smoking with or without liver fibrosis.

Variables	Nonalcoholic fatty liver disease with smoking		*P*
	Fibrosis	Nonfibrosis	
Sample size	46	52	
Sex (F/M)	1/45	2/50	0.632
Age (years)	48.78 ± 11.65	43.09 ± 13.92	0.032
Height (cm)	168.17 ± 6.51	168.52 ± 6.49	0.794
Weight (kg)	67.37 ± 13.57	65.79 ± 11.56	0.535
SBP (mm/Hg)	125.71 ± 14.25	128.17 ± 10.82	0.337
DBP (mm/Hg)	80.76 ± 8.92	83.21 ± 6.64	0.124
ALT	87.91 ± 82.58	72.12 ± 81.30	0.344
AST	83.78 ± 38.15	53.74 ± 46.76	0.001
PLT	201.55 ± 70.21	228.99 ± 55.43	0.036
T2DM (Y/N)	14/32	7/45	0.041
Smoking index	583.26 ± 480.72	388.63 ± 458.52	0.043

**Table 5:** Risk factors for fibrosis in NAFLD patients with smoking.

Variables	Univariate analysis			Multivariate analysis		
	OR	95% CI	*P*	OR	95% CI	*P*
Sex	1.022	0.395–1.895	0.614			
Age	1.049	1.004–1.095	0.029			
Height	0.956	0.867–1.054	0.983			
Weight	1.069	1.007–1.136	0.176			
SBP	1.036	0.943–1.138	0.113			
DBP	0.870	0.747–1.014	0.076			
ALT	0.997	0.991–1.004	0.310			
AST	1.024	1.008–1.040	0.004	1.040	1.004–1.078	0.029
PLT	0.991	0.982–1.000	0.010			
Smoking index	1.666	1.187–2.338	0.014	1.220	1.040–1.878	0.019
T2DM	1.199	1.036–3.991	0.011	1.054	1.067–3.050	0.032

## Data Availability

The data used to support the findings of this study are available from the corresponding author upon request.
